# Endoscopic Sphincterotomy with Large Balloon Dilation versus Endoscopic Sphincterotomy for Bile Duct Stones: A Systematic Review and Meta-Analysis

**DOI:** 10.1155/2015/673103

**Published:** 2015-02-10

**Authors:** Lei Xu, Moe Htet Kyaw, Yee Kit Tse, James Yun Wong Lau

**Affiliations:** ^1^Department of Gastroenterology, Ningbo No. 1 Hospital, Ningbo 315010, China; ^2^Institute of Digestive Diseases, Prince of Wales Hospital, The Chinese University of Hong Kong, Shatin, Hong Kong; ^3^Department of Surgery, Prince of Wales Hospital, The Chinese University of Hong Kong, Shatin, Hong Kong

## Abstract

The safety and efficacy of endoscopic sphincterotomy with large balloon dilation (EPLBD) are unclear. This study compares the safety and efficacy between EPLBD and endoscopic sphincterotomy (EST).* Patients and Methods*. Literatures were searched for randomized controlled trials in PUBMED, EMBASE, and Cochrane Library. Outcome measurements included adverse events; stone removal rate; requirement of mechanical lithotripsy.* Results*. Four RCTs with a total of 596 patients were included. Three RCTs compared EPLBD versus EST alone for stone removal; one RCT compared EPLBD versus EST plus mechanical lithotripsy for stone removal. Pooled data from three RCTs showed that there was no significant difference in the adverse event of ERCP. A significantly higher cholangitis rate was seen in patients who received EST plus mechanical lithotripsy, compared to those treated with EPLBD (13.3% versus 0.0, *P* = 0.026). No statistical difference was found between EPLBD and EST for stone removal rate. Significant differences in requirement of mechanical lithotripsy were seen with removal of large stones (>15 mm), with EPLBD reducing the use of mechanical lithotripsy (RR: 0.73; 95% CI: 0.54–0.99).* Conclusions*. EPLBD and EST have similar efficacy and safety for bile duct stones clearance. With larger stones, EPLBD can reduce requirement of mechanical lithotripsy.

## 1. Introduction

During endoscopic retrograde cholangiopancreatography (ERCP), the removal of common bile duct stones involves the destruction or dilatation of the bile duct orifice. Endoscopic sphincterotomy (EST) has been the standard method of management for removal of stones from the common bile duct (CBD) since it was described in 1974 [1]. However, when faced with more challenging situations, for example, in patients with large or multiple stones and tapered or tortuous distal common duct, additional techniques such as mechanical lithotripsy may be utilized [2, 3]. Furthermore, EST and stone removal can result in adverse events, including bleeding, pancreatitis, perforation, and cholangitis [4, 5].

As an alternative method to EST, endoscopic papillary balloon dilation (EPBD) was described by Staritz et al. [6] for the management of CBD stones. Compared to EST, EPBD has been reported to have a lower risk of bleeding but an increased risk of post-ERCP pancreatitis (PEP) [7]. Additionally, removal of large stones may be challenging when using EPBD alone. Thus, in 2003, Ersoz et al. [8] modified the technique of EPBD by introducing EST prior to large balloon dilatation for the removal of large bile duct stones, which has now been described as endoscopic sphincterotomy with large balloon dilation (EPLBD). Since then, studies comparing the efficacy and safety of EPLBD with EST have reported mixed outcomes. A previous meta-analysis conducted by Feng et al. comparing EPLBD with ES showed EPLBD to have fewer overall adverse events [9]. However, the meta-analysis included pooling of nonrandomized and retrospective studies, which may have induced bias. Furthermore, additional studies have now been reported in the literature.

We performed a systematic review and meta-analysis to clarify the safety and efficacy of EPLBD and EST. Compared to existing meta-analysis, we have only included randomized controlled trials.

## 2. Methods

### 2.1. Inclusion Criteria

We defined inclusion criteria according to the PICOS [10]: (1) participants (P): all the patients with bile duct stones who received ERCP, (2) interventions (I) and comparisons (C): comparing sphincterotomy plus large balloon dilation (EPLBD) versus endoscopic sphincterotomy (EST), (3) outcomes (O): the primary outcome being the overall adverse event rate of EPLBD compared with EST and the secondary outcomes including individual adverse event rates (including post-ERCP pancreatitis (PEP), hemorrhage, biliary tract infection, and perforation), stone removal rate (including stone clearance rate at the first session of ERCP, large stone clearance rate at the first session of ERCP, and the total stone clearance rate in all the session of ERCP), and the requirement of mechanical lithotripsy, and (4) study design (S): randomized controlled trials (RCTs).

### 2.2. Search Strategy

An electronic search was performed using the keyword combined with medical subject headings (MeSH). We searched full publications and abstracts from the following computerized databases: MEDLINE, EMBASE, and the Cochrane Central Register of Controlled Trials in the Cochrane Library (1981–2013). The search was limited to clinical trials and articles published in English.

### 2.3. Data Extraction

To avoid discordant evaluation and extractor bias, two analysts performed two separate readings (Lei Xu and Moe Htet Kyaw). Discrepancies between the two investigators were resolved by discussion and consensus with a senior investigator (James Yun Wong Lau).

### 2.4. Risk of Bias Assessment

The quality of the included studies was assessed according to the Cochrane Collaboration's tool for assessing risk of bias of RCTs [11]. The risk domains of assessment included sequence generation, allocation concealment, blinding of participants and personnel, incomplete outcome data, selective reporting, and other sources of bias.

### 2.5. Statistical Analysis

Statistical analyses were performed by RevMan software (Review Manager Version 5.2, the Nordic Cochrane Centre, Copenhagen, Denmark). For dichotomous variables compared within each trial, risk ratio (RR) and 95% confidence intervals (95% CI) were calculated. Statistical heterogeneity among trials was evaluated by Cochrane Q with chi-squared test, and *P* < 0.1 was considered to be significant heterogeneity. Quantity of statistical heterogeneity was assessed with *I* ^2^. With higher values of *I* ^2^ showing increasing heterogeneous studies, a random-effect model was applied. In the condition of clinical heterogeneity in the therapeutic methods, pooling of data was not performed, and the results were further evaluated with subgroup analyses.

## 3. Results

### 3.1. Study Characteristics

**Figure 1:**
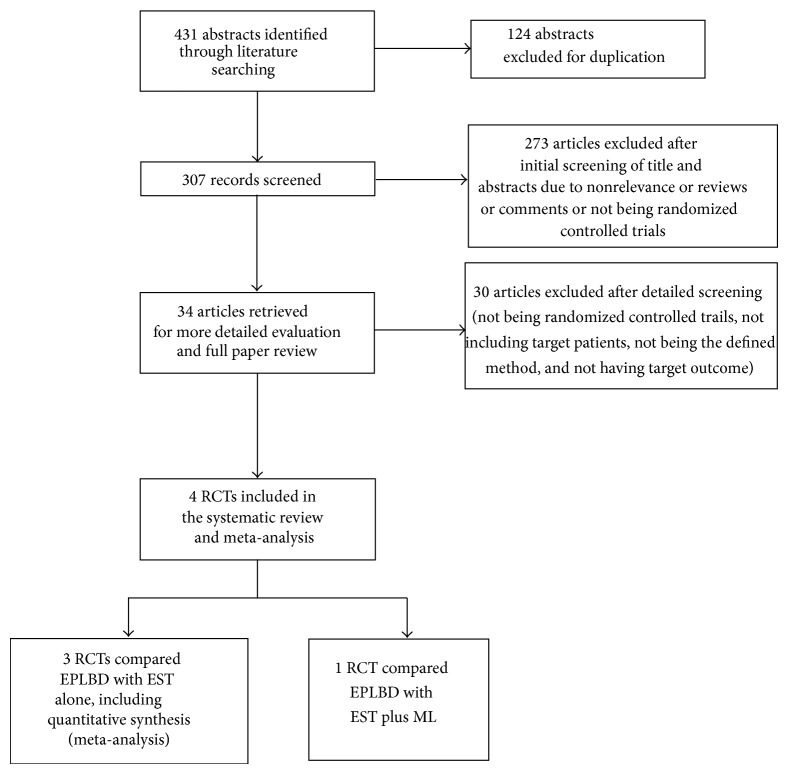
Flow diagram on literature search. EPLBD: endoscopic sphincterotomy with large balloon dilation; EST: endoscopic sphincterotomy.

The initial search identified 431 potential abstracts. The title and abstract were reviewed, 397 studies were rejected because of duplications, nonrelevance, or reviews or comments or not being randomized controlled trials. 34 articles were retrieved for more detailed evaluation and full paper review. 30 articles were excluded because of nonrandomization, not including the target patients, not having defined method, not having the target outcomes. The final meta-analysis included four studies (Figure 1). Among the four RCTs, three RCTs compared EPLBD versus EST alone for stone removal [12–14], and one RCT compared EPLBD versus EST plus mechanical lithotripsy for stone removal [15]. Because the intervention in the study by Stefanidis et al. was different from the other three studies, the study was excluded in the pooled analysis [15].

A total of 496 individuals fulfilled our inclusion criteria.  Table 1 lists the characteristics of the patients in the 4 included studies. Overall, 245 patients were randomized to EPLBD and 251 patients were randomized to EST. In all studies, the size of the stone or common bile duct (CBD) was reported to be bigger than 12 mm. The balloon size used for dilatation ranged from 12 mm to 20 mm. With the exception of the study by Stefanidis et al., the sphincterotomy of the patients randomized to EPLBD was less than half of the papilla. This was followed by balloon dilation. For patients randomized to EST alone, a complete sphincterotomy was performed. In the study by Stefanidis et al., EST was completed to its full length in both groups. This was carried out by extending the incision up to but not through the major horizontal fold crossing the intramural portion of the bile duct.

### 3.2. Risk of Bias

**Figure 2:**
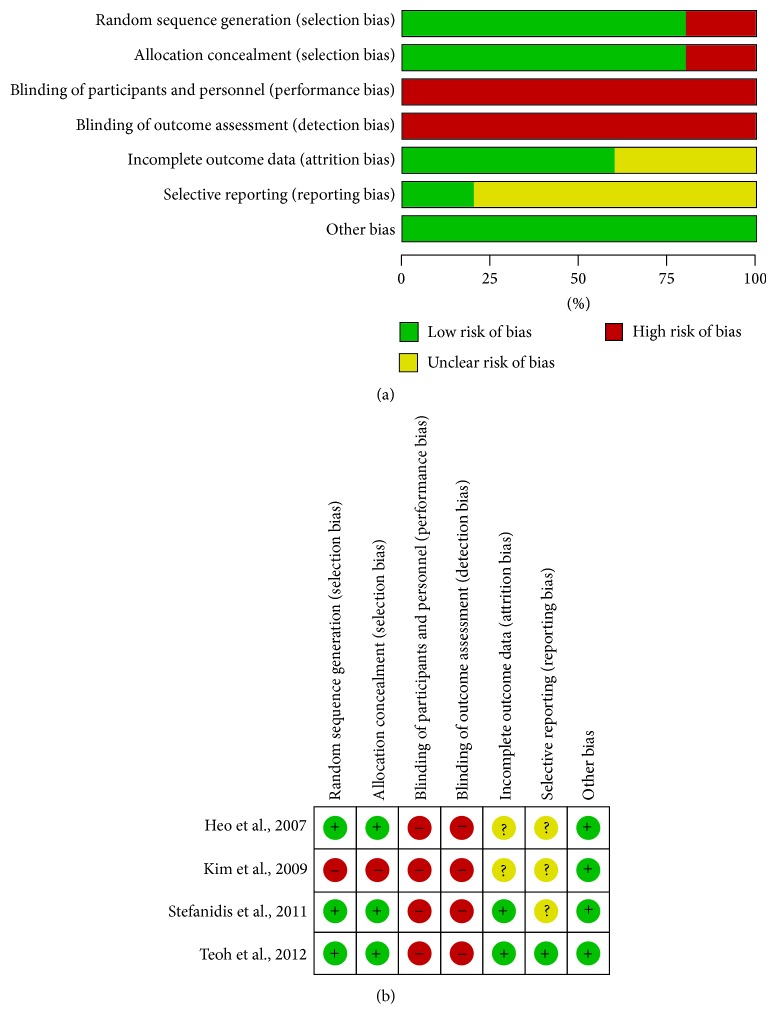
(a) Risk of bias graph. (b) Risk of bias summary.

Among these 4 RCTs, no study reported the use of blinding. All but one presented description of random sequence generation and allocation concealment. Two of the included studies had low risk of bias for incomplete data and one for selective reporting (Figure 2).

#### 3.2.1. Overall Adverse Event

The adverse events were defined according to consensus guidelines, including post-ERCP pancreatitis (PEP), hemorrhage, biliary tract infection, and perforation. We analyzed the differences in the overall adverse event rate between the two interventions as the primary outcome.

**Figure 3:**
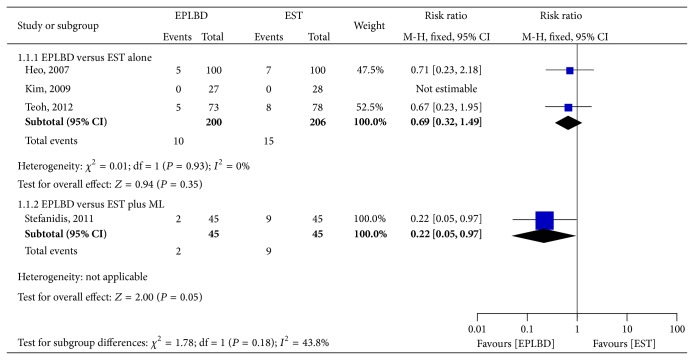
Forest plot on the overall adverse events comparing EPLBD versus EST.

Pooled data from three RCTs did not show statistical differences in the overall adverse event rates between EPLBD and EST alone for clearance of CBD stones (5.0% versus 7.3%, resp.; RR 0.69; 95% CI: 0.32–1.49) [12–14]. In the RCT comparing EPLBD with EST plus mechanical lithotripsy, EPLBD had less adverse events (4.4% versus 20%, *P* = 0.049) [15] (Figure 3).

#### 3.2.2. Secondary Outcome

*(1) Adverse Event*. In the 3 RCTs comparing EPLBD with EST alone, there were no statistical differences in the each form of adverse event: PEP (3.0% versus 3.4%, resp.; RR 0.88; 95% CI: 0.30–2.57); hemorrhage (0.5% versus 1.0%, resp.; RR 0.69; 95% CI: 0.12–4.01); biliary tract infection (1.5% versus 1.9%, resp.; RR 0.79; 95% CI: 0.18–3.45); perforation (0% versus 1.0%, resp.; RR 0.21; 95% CI: 0.01–4.37).

**Figure 4:**
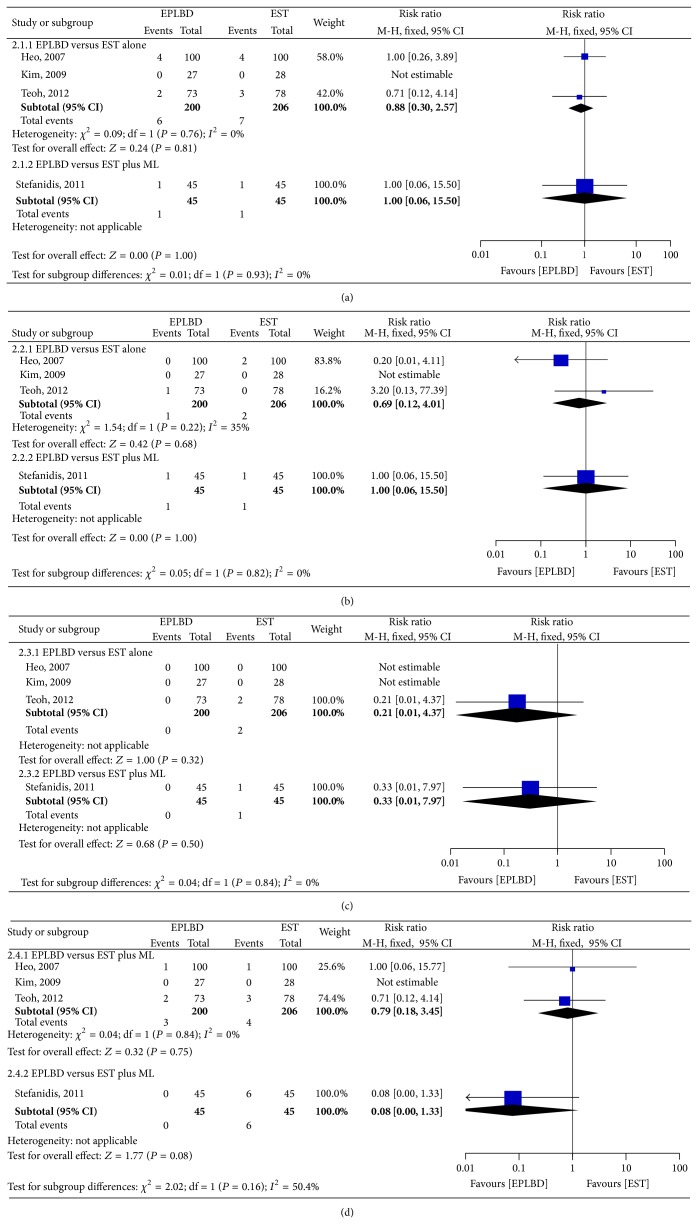
Forest plot on the adverse events compared EPLBD versus EST: (a) pancreatitis, (b) bleeding, (c) perforation, and (d) biliary tract infection.

In the RCT comparing EPLBD with EST plus ML, cholangitis rate was higher in the patients receiving EST plus mechanical lithotripsy compared to EPLBD (13.3% versus 0.0, *P* = 0.026). No statistical differences were found in the other forms of adverse events between EPLBD and EST plus ML (Figure 4).

**Figure 5:**
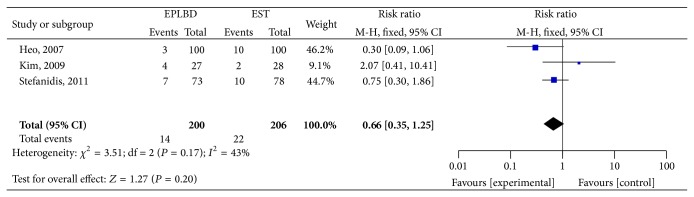
Forest plot on the endoscopic bleeding compared EPLBD versus EST.

Endoscopic bleeding was measured in all four studies [12–15]. From the pooled data of three RCTs that reported bleeding, there was no statistical difference found between EPLBD and EST (7% versus 10.6%, resp.; RR 0.66; 95% CI: 0.35–1.25). In the RCT that comparing EPLBD with EST plus mechanical lithotripsy, no intraprocedural bleeding was seen in any of the patients (Figure 5).

**Figure 6:**
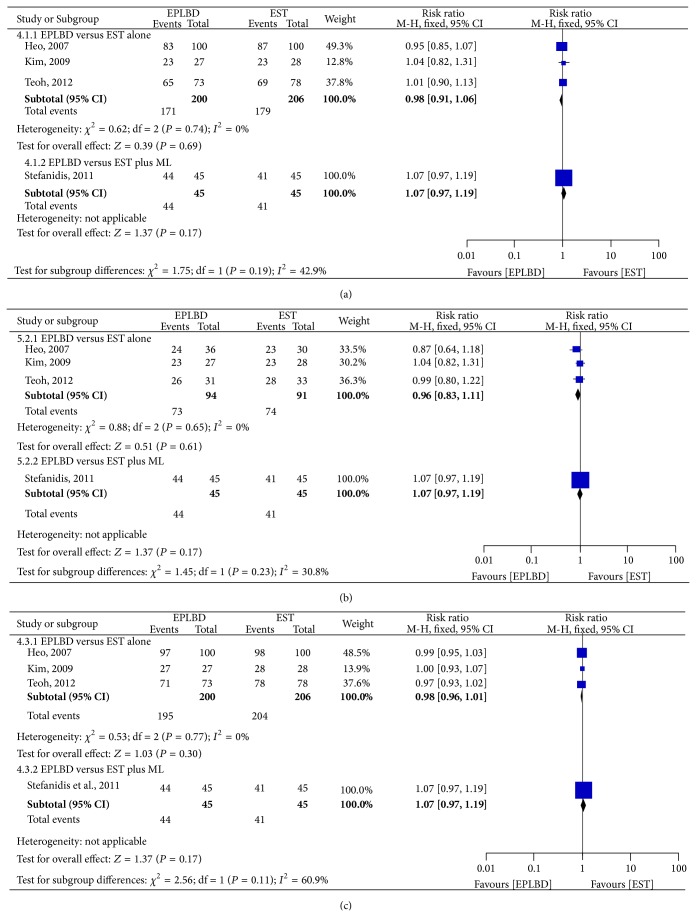
Forest plot on the stone removal rate compared EPLBD versus EST: (a) stone clearance rate at the first session of ERCP, (b) total stone clearance rate, and (c) large stone clearance rate at the first session of ERCP.

*(2) Stone Removal Rate*. All four studies compared the stone clearance rate in the first session. A total of 406 patients were enrolled in three RCTs that compared EPLBD and EST alone; 90 patients were enrolled in one RCT that compared EPLBD and EST plus mechanical lithotripsy. Each individual study showed no statistical differences between the EPLBD and EST in stone clearance rate. After pooled data of three RCTs, there were no statistical differences between the EPLBD and EST alone for removal of CBD stones in the first session (85.5% versus 86.9%, resp.; RR 0.98; 95% CI: 0.91–1.06) (Figure 6(a)).

The three RCTs also compared the large stone clearance rate at the first session of ERCP and the total stone clearance rate in all the sessions. For stones larger than 15 mm, there was no statistical differences between two groups in terms of stone clearance rate in the first session (77.7% versus 81.3%, resp.; RR 0.96; 95% CI: 0.83–1.11). No statistical difference was seen for the total stone clearance rate in all sessions (97.5% versus 99.0%, resp.; RR 0.98; 95% CI: 0.96–1.01) (Figures 6(b) and 6(c)).

In the study comparing EPLBD with EST plus mechanical lithotripsy, no statistical difference was found in the clearance rate of large stones (defining stone bigger than 12 mm) (Figure 6).

**Figure 7:**
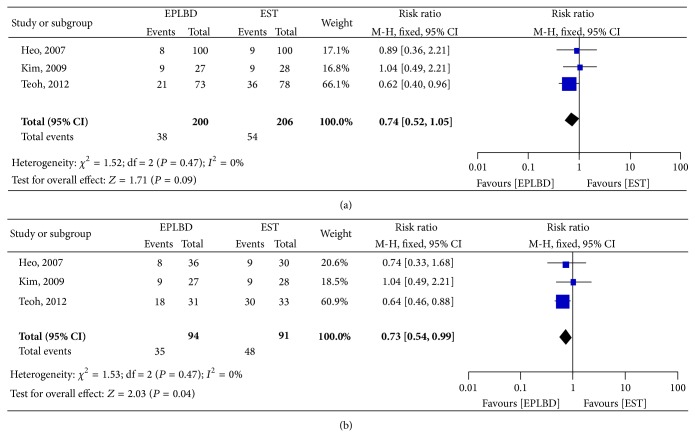
Forest plot on the mechanical lithotripsy using rate compared EPLBD versus EST: (a) all stones; (b) stones bigger than 15 mm.

*(3) Mechanical Lithotripsy Requirement Rate*. Three studies were identified for comparing mechanical lithotripsy requirement between EPLBD and EST alone for all stones and stones bigger than 15 mm. One study showed that EPLBD reduced the need for mechanical lithotripsy [14], while two other studies showed similar rate of mechanical lithotripsy requirement [12, 13]. Pooled date of three studies showed no statistical differences between the two groups (19% versus 26.2%; RR 0.74; 95% CI: 0.52–1.05). For the use of mechanical lithotripsy in large stones (more than 15 mm), one study showed that EPLBD reduced the need for mechanical lithotripsy [14], while two studies showed similar rate of mechanical lithotripsy requirement [12, 13]. Pooled data of three studies showed that EPLBD significantly reduced the use of mechanical lithotripsy in clearance of large stones (37.2% versus 52.7%; RR 0.73; 95% CI: 0.54–0.99) (Figure 7).

## 4. Discussion

Our meta-analysis evaluated randomized controlled trials comparing endoscopic sphincterotomy with large balloon dilation (EPLBD) and endoscopic sphincterotomy (EST). After pooled analysis, there was no significant difference in the overall adverse event rate between EPLBD and EST. In addition, there was no significant difference with individual forms of adverse events between the two interventions, such as post-ERCP pancreatitis (PEP), hemorrhage, infection, and perforation. Pooled analysis showed no difference in stones clearance rate. However, with removal of large stones (more than 15 mm), the requirement of ML was less than that for patients that received EPLBD compared to those that received EST.

In 10–15% of patients, it may be difficult to remove stones using EST and conventional methods. Such difficulties may be related to several factors: large stones, barrel-shaped stones, tapering of the distal common duct, and so forth [2, 3]. The additional methods including mechanical lithotripsy, shock wave, mother-baby laser, or electrohydraulic lithotripsy may be applied in such circumstances [2]. Ersoz et al. [8] developed the EPLBD (≥12 mm) as an alternative method to EST to deal with difficult bile duct stones. Subsequent studies using EPLBD showed that the overall stones clearance rate was 83–100% [12–14, 16–20]. Previous systematic reviews and meta-analysis showed that endoscopic balloon dilatation and EST have similar efficacy of stone clearance [7, 21]. However, such analysis included nonrandomized studies.

From our meta-analysis involving RCTs only, the stones clearance rates using EPLBD were as follows. The stone clearance rate for all stone sizes (≥12 mm) in the first session was 87.8%. The stone clearance rate for larger stones (≥15 mm) in the first session was 77.7%. The total stone clearance rate in all the sessions was 97.5%. In comparison with EST, there was no significant difference in stone clearance rate. It is worth noting that 3 studies [12–14] described the methods of EPLBD as minor or mid-EST plus LBD. It is often difficult to measure length of sphincterotomy in clinical endoscopic procedure. The study by Stefanidis [15] had a different study design compared to other three studies, thus this study was not added to the pooled analysis.

All studies compared the adverse events between the two methods. Three studies defined the adverse event according the consensus guideline [4, 22, 23]; one defined it according to a similar alternative guideline [23–25]. Endoscopists are concerned with the risk of PEP related to endoscopic balloon dilation (ESBD). One study reported a higher incidence of PEP with two subsequent deaths in the ESBD group (2/85) [26]. A report from two meta-analyses showed that endoscopic balloon dilation has a higher risk of inducing pancreatitis compared with EST [7, 27]. This led to strategies to modify methods of ESBD to reduce the risks of PEP. Another subsequent meta-analysis of three RCTs and six nonrandomized controlled trials showed that ESBD did not increase the risks of PEP. This meta-analysis also included studies using EPLBD in addition to ESBD [9]. Similar results were shown in our meta-analysis. The potential reason for a low risk of PEP after EPLBD may be explained by the additional effect of sphincterotomy prior to balloon dilatation. The sphincterotomy may direct the force exerted by the dilating balloon toward the CBD instead of the pancreatic orifice.

Bleeding is one of the most common severe adverse events of ERCP. In theory, with larger area of tissue damage, large balloon dilation would increase the risks of bleeding compared to conventional balloon. But our meta-analysis showed that bleeding rate was low in both interventions, with a lower rate in EPLBD (2/245, 0.8%) compared to EST (3/251, 1.2%). However, no statistical difference was found between the two groups. With a small number of RCTs and all reported conflicting results, we should be very careful towards our conclusion. Further studies are required to clarify the bleeding risk of EPLBD. We should also consider other factors that may increase risk of bleeding, such as tapered distal bile duct [8].

Perforation during balloon dilatation is uncommon, but it can present as a severe and fatal adverse event of ERCP [22, 23]. A case report has highlighted the possibility of cystic duct perforation after balloon overinflation [20]. In our meta-analysis, two guide wire perforations were recorded in the EST group [14]; one main bile duct perforation was recorded in the EST group [15]. However, the perforations reported were unlikely to be associated with sphincterotomy. In all four studies, no perforation was recorded in patients that received EPLBD. No statistical difference with the risk of perforation was found between the two interventions.

The incidence rate of biliary tract infection was rare among the 3 RCTs that compared EPLBD with EST alone, with no significant differences found between the two interventions (3/200, 1.5%, versus 4/206, 1.9%). However, in the study by Stefanidis et al. [15], which compared EPLBD with EST plus mechanical lithotripsy, a higher cholangitis rate was observed in the latter group (0/45 versus 6/45, 13.3%). The reason for a higher cholangitis rate may be explained by the following: trauma to bile duct wall by the lithotripter wire; inadequate sphincterotomy; edema at the sphincterotomy site.

Whether EPLBD could reduce the need of mechanical lithotripsy is another important clinical question. A number of case series have suggested that EPLBD may be useful in reducing the need for ML [16–19, 28, 29]. In the three RCTs included in our meta-analysis, only the study by Teoh et al. showed EPLBD to reduce the need of mechanical lithotripsy [14]. While the other two studies showed similar requirement of mechanical lithotripsy for clearance of stones of all sizes. [12, 13]. For the removal of large stones (lager than 15 mm), pooled analysis showed that the requirement rate of mechanical lithotripsy was less using EPLBD compared to EST alone. Theoretically, a large orifice after EPLBD makes removal of large stones easier compared to EST alone and reduced the requirement of ML. Reduction in the use of mechanical lithotripsy means less risk of trauma to bile duct by the lithotripter wires and more cost savings.

## 5. Limitations

There are several limitations in this study. First, the number of patients included in this study is small. With rare adverse events of ERCP, further larger studies are required to assess the safety of the two methods. Second, EPLBD is commonly used for the removal of large stones with dilated CBD. However, only 2 studies [13, 15] of the 4 RCTs was pooled 100% large stones and the other two papers [12, 14] included certain percentage of small stones. More RCTs focusing on large stones are required to assess the precise benefit of EPLBD. Third, for patients with fibrotic stricture at distal CBD or ampulla tissue, the EPLBD was not indicated, while the 4 RCTs did not mention the assessment of the stricture, which may cause bias. Fourth, clinical heterogeneity existed in individual studies. The study by Stefanidis had a different study design compared to other three studies; thus, this study was not added to the pooled analysis. Other sources of heterogeneity that may have result in bias include the following. The presence of periampullary diverticulum was not reported in all studies. The description of the length of EST varied between the studies. The balloon inflating time and balloon diameter reported in the each study were also different, and this may affect the overall risk of pancreatitis and bleeding [30]. The indication of mechanical lithotripsy may be different for the individual endoscopists. This may explain the higher rate of ML in the study by Teoh et al. compared to the other studies. Endoscopists could not be blinded after the dilation or sphincterotomy was randomly assigned. Because of the nature of the intervention, such bias could not be avoided. Additionally, our search strategy included articles only published in English; articles published in other languages were not included because of anticipated difficulties in obtaining accurate medical translation.

## 6. Conclusion

In the removal of bile duct stones, EPLBD and EST have similar safety and efficacy. However, with clearance of larger stones, EPLBD can reduce the requirement of mechanical lithotripsy, which may reduce the risk of cholangitis and save cost. Further studies comparing the cost effectiveness of the two interventions are required.

## Figures and Tables

**Table 1:** Baseline characteristics comparing endoscopic sphincterotomy with large balloon dilation (EPLBD) versus endoscopic sphincterotomy (EST).

Trials	Site	Number of patients		Stone size (diameter, mm)		EPLBD procedure		The length of the sphincterotomy	
		EPLBD	EST	Range	Mean	Balloon size (mm)	balloon inflated time	EPLBD	EST
Heo et al., 2007 [12]	Korea	100	100	≤40	16.0 ± 0.7 (EPLBD) versus15.0 ± 0.7 (EST)	12–20	60 seconds	Limited to a third size of full length	Complete sphincterotomy
Kim et al., 2009 [13]	Korea	27	28	15–50	20.8 ± 4.1 (EPLBD) versus 20.5 ± 5.7 (EST)	15, 16.5, and 18	Not mentioned	Midportion of the papilla	Complete sphincterotomy
Teoh et al., 2013 [14]	Hong Kong	73	78	Not mentioned	12.47 (5–35) (EPLBD) versus 13.26 (5–40) (EST)	13–15	30 seconds	One-third to one-half of the papilla	Complete sphincterotomy
Stefanidis et al., 2011 [15]	Greece	45	45	12–20	Not mentioned	15, 18, and 20	10–20 seconds	Complete sphincterotomy	Complete sphincterotomy
